# Author Correction: A natural mutation in the promoter of *Ms-cd1* causes dominant male sterility in *Brassica oleracea*

**DOI:** 10.1038/s41467-024-52884-4

**Published:** 2024-10-01

**Authors:** Fengqing Han, Kaiwen Yuan, Wenru Sun, Xiaoli Zhang, Xing Liu, Xinyu Zhao, Limei Yang, Yong Wang, Jialei Ji, Yumei Liu, Zhansheng Li, Jinzhe Zhang, Chunzhi Zhang, Sanwen Huang, Yangyong Zhang, Zhiyuan Fang, Honghao Lv

**Affiliations:** 1 State Key Laboratory of Vegetable Biobreeding, Institute of Vegetables and Flowers, Chinese Academy of Agricultural Sciences, Beijing, 100081 China; 2 Key Laboratory for Vegetable Biology of Hunan Province, Engineering Research Center for Horticultural Crop Germplasm Creation and New Variety Breeding, Ministry of Education, Hunan Agricultural University, Changsha, 410128 China; 3 State Key Laboratory of Vegetable Biobreeding, Tianjin Academy of Agricultural Sciences, 300192 Tianjin, China; 4 Shenzhen Branch, Guangdong Laboratory of Lingnan Modern Agriculture, Genome Analysis Laboratory of the Ministry of Agriculture and Rural Affairs, Agricultural Genomics Institute at Shenzhen, Chinese Academy of Agricultural Sciences, Shenzhen, Guangdong 518120 China; 5 Chinese Academy of Tropical Agricultural Sciences, Haikou, Hainan 571101 China

Correction to: *Nature Communications* 10.1038/s41467-023-41916-0, published online 05 October 2023

In the original version of this Article, the image of wild-type (WT) 01-20 anther at stage 7 (S7) presented in Fig 1c was incorrectly chosen from the images taken from the same development stage of the same genotype but different batch of experiment for Fig. 5f. It has been replaced by the correct representative image in both the PDF and HTML versions of the Article.

