# Supplementary material for: A natural mutation in the promoter of Ms-cd1 causes dominant male sterility in Brassica oleracea
Source: Nat Commun. 2023 Oct 5;14:6212. doi: 10.1038/s41467-023-41916-0 (PMC10556095; doi:10.1038/s41467-023-41916-0)
Supplement: Supplementary file 1 — Supplementary Information [file 41467_2023_41916_MOESM1_ESM.pdf]

A natural mutation in the promoter of *Ms-cd1* causes dominant male sterility in *Brassica oleracea*

Han *et al.*

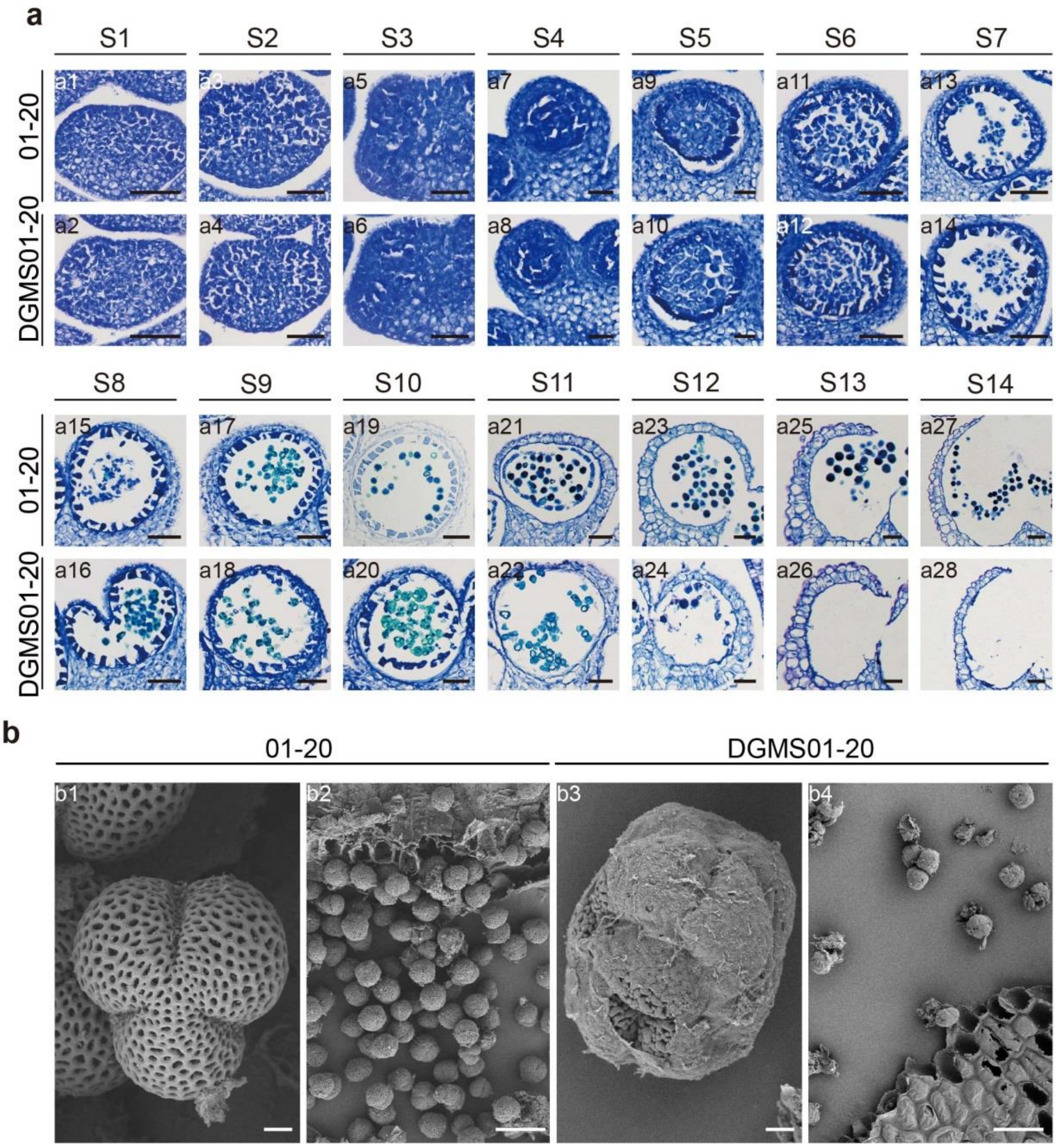

Supplementary Figure 1. Transverse section and SEM analyses of wild-type 01-20 and DGMS01-20 mutant. (**a**) Anther development in cabbage is divided into 14 stages (S1-S14) in wild-type 01-20 and the corresponding stages in DGMS01-20. Scale bar, 20 μm for stages S1-S5, 50 μm for stages S6-S14. (**b**) SEM analyses of microspore in wild-type 01-20 and DGMS01-20. Scale bar, 3 μm in b1 and b3, 50 μm in b2 and b4. Experiments were repeated three times independently with similar results.

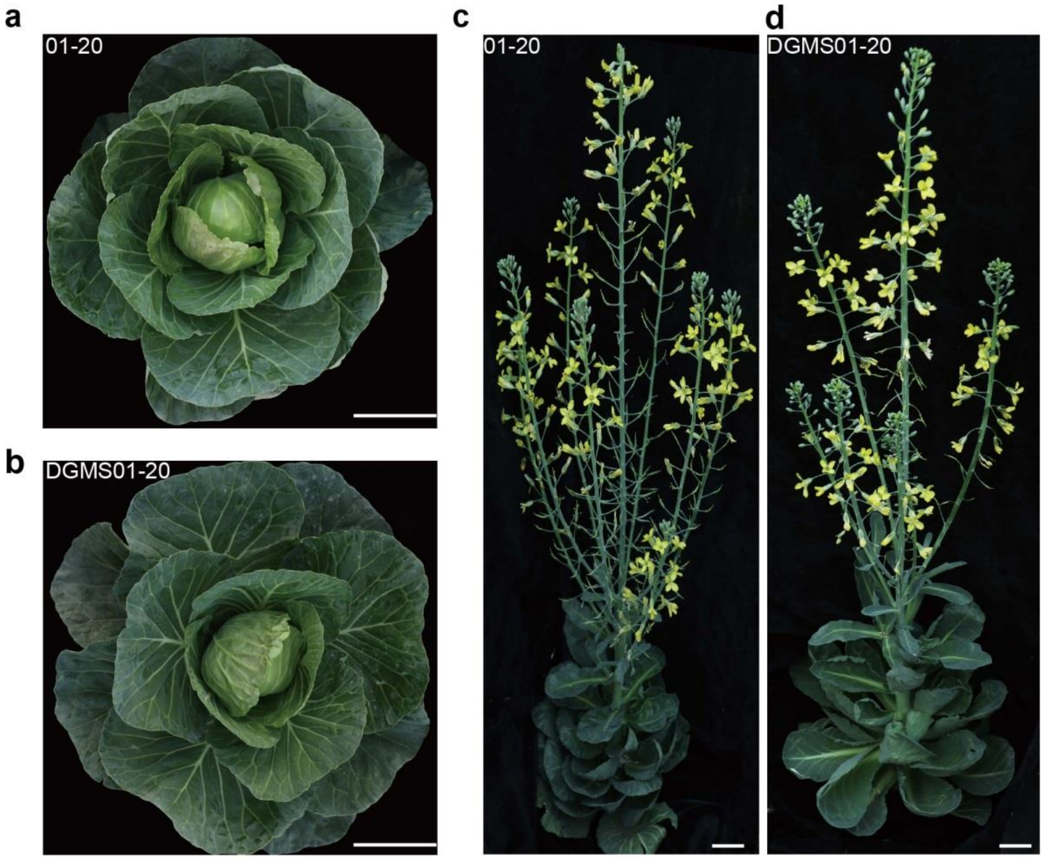

Supplementary Figure 2. Comparation of whole plant phenotypes of wild-type 01-20 and DGMS01-20 mutant. (**a** and **b**) 01-20 and DGMS01-20 plants and heading stage. Scale bar, 15 cm. (**c** and **d**) 01-20 and DGMS01-20 plants and flowering stage. Scale bar, 5 cm.

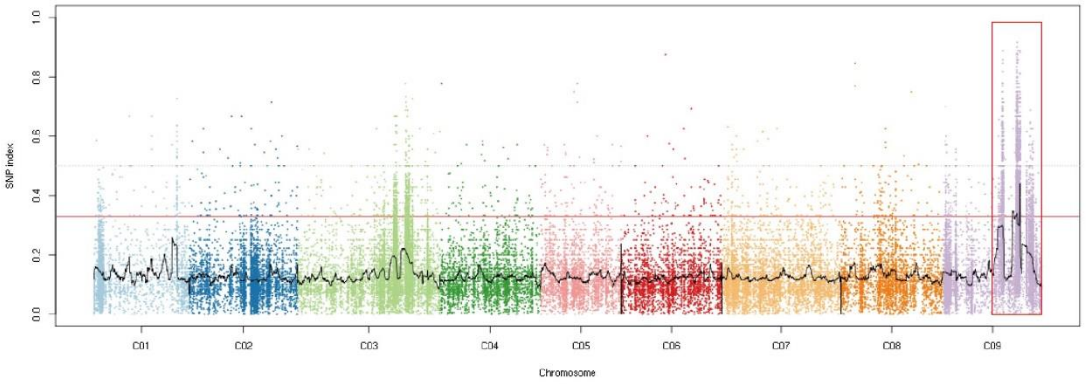

Supplementary Figure 3. ***Ms-cd1*** **was mapped on chromosome 9 based on BSA-seq using PO1.**

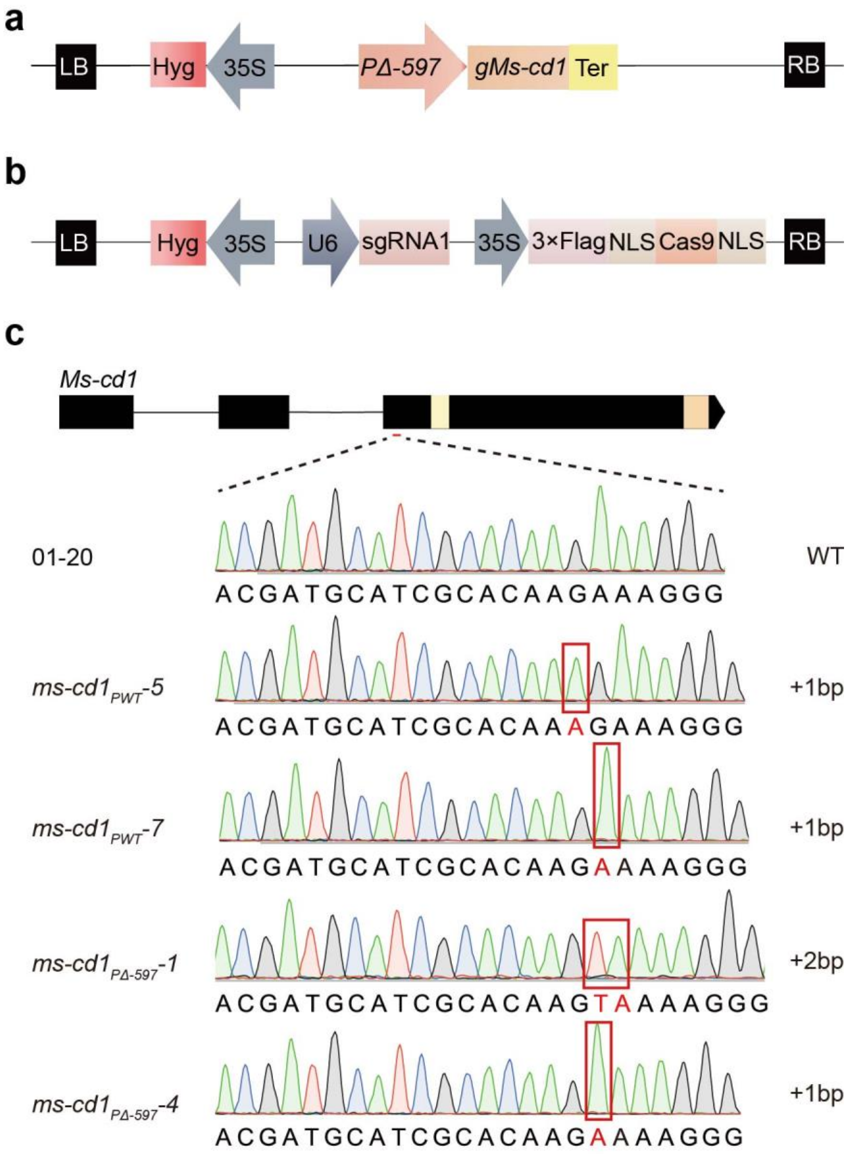

Supplementary Figure 4. Schematic diagram of the complementation construct *PΔ-597::gMs-cd1*, CRISPR/Cas9 construct, *Ms-cd1* gene structure, and representative plants edited at the target region of *Ms-cd1*. (**a**) Schematic diagram of the complementation construct *PΔ-597::gMs-cd1*. *HYG*, *hygromycin*-resistance gene; 35S, CaMV 35S promoter. (**b**) Schematic diagram of the CRISPR/Cas9 construct targeting *Ms-cd1*. U6, *Arabidopsis* U6-26 promoter; sgRNA1, a single guide RNA targeting *Ms-cd1*. (**c**) Gene structure of *Ms-cd1*, and DNA sequence analysis of the sgRNA targeted site among WT and the four knock-out lines generated by CRISPR/Cas9 system. Mutations are indicated in red. The putatively conserved LXXLL motif and PHD domain of *Ms-cd1* are indicated by blocks.

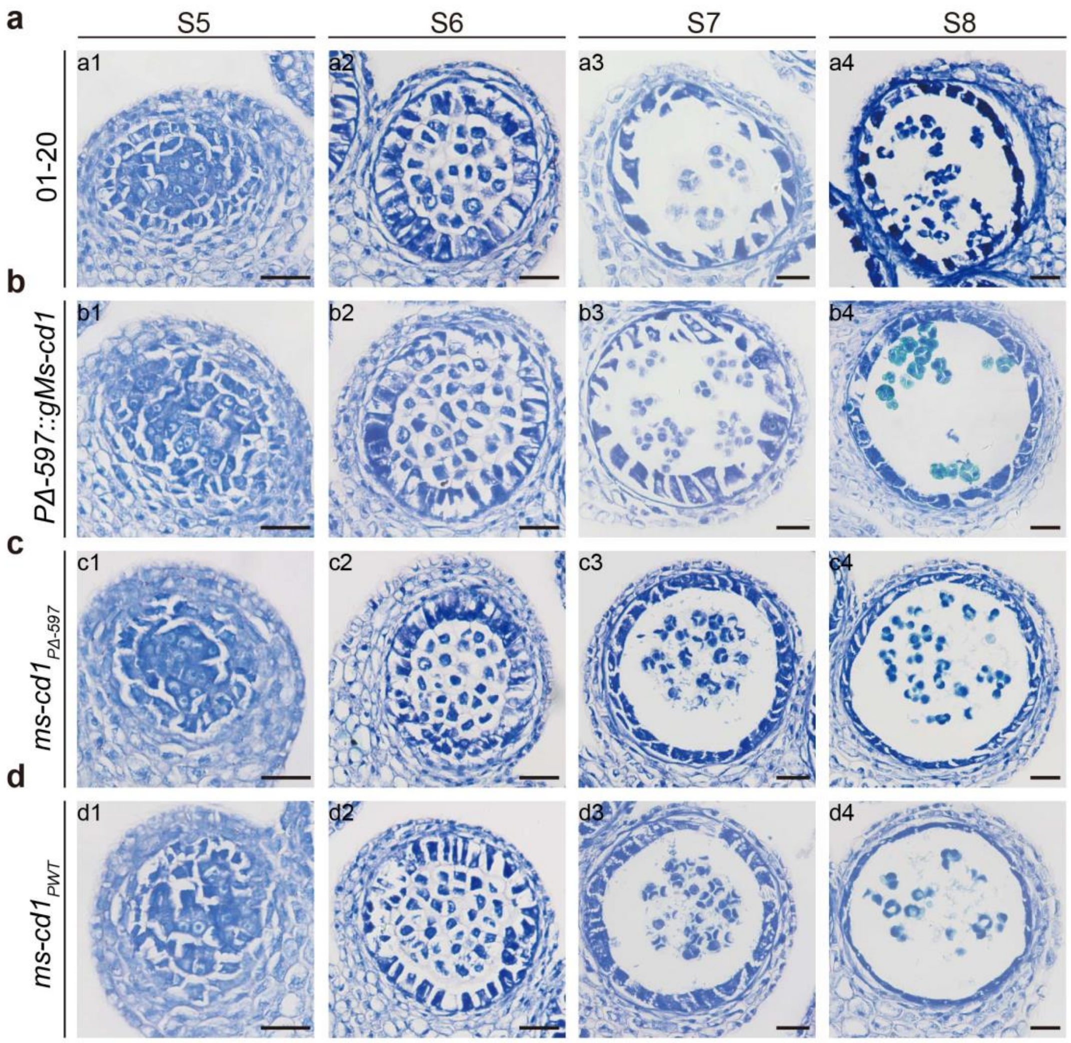

Supplementary Figure 5. Transverse section analyses of anthers in WT, *PΔ-597::gMs-cd1*, *ms-cd1*$_{PWT}$ and *ms-cd1*$_{PΔ\text{-}597}$ mutants at anther development stages S5-S8. (a) Representative anther transverse sections at stages S5-S8 in wild-type 01-20. **(b)** Representative anther transverse sections at stages S5-S8 in *PΔ-597::gMs-cd1* transgenic plants. **(c)** Representative anther transverse sections at stages S5-S8 in *ms-cd1*$_{PΔ\text{-}597}$ mutant. **(d)** Representative anther transverse sections at stages S5-S8 in *ms-cd1*$_{PWT}$ mutant. Scale bars, 25 μm. Experiments were repeated three times independently with similar results.

a

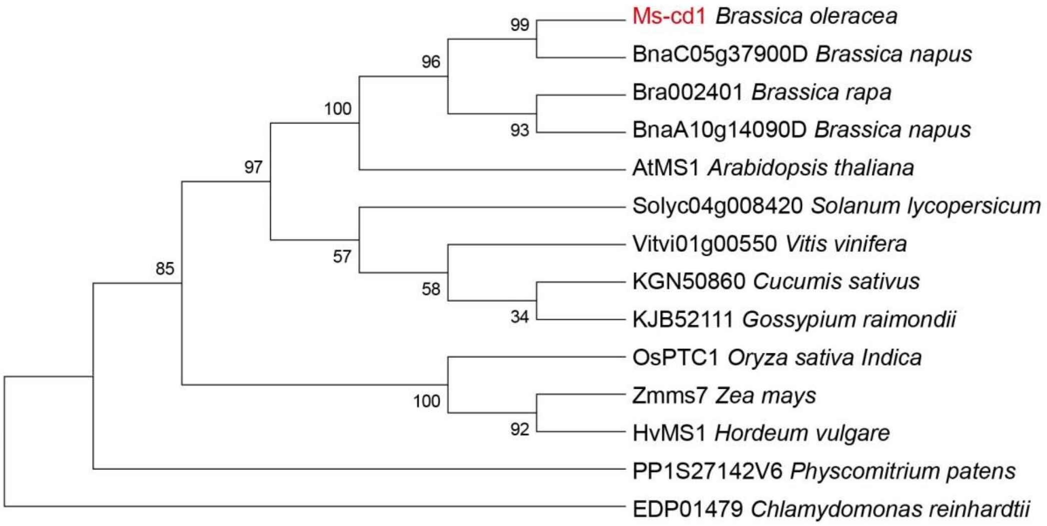

b

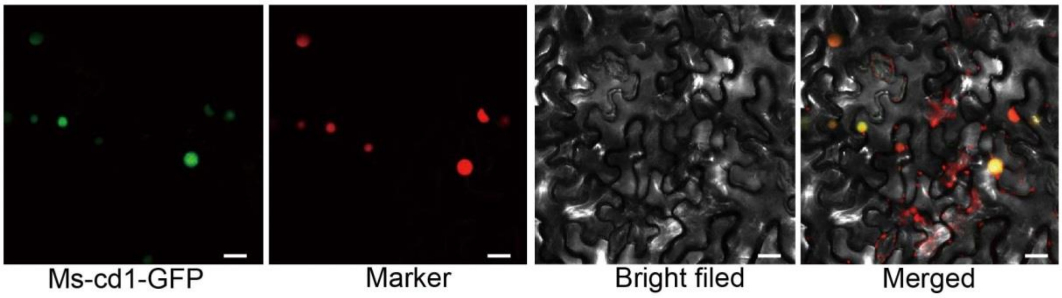

Supplementary Figure 6. Phylogenetic analysis and subcellular localization of Ms-cd1. (**a**) Phylogenetic analysis of Ms-cd1 homologues in 13 species. Ms-cd1 is indicated in red. The neighbour-joining phylogenetic tree (1000 bootstrap replications) was constructed using MEGA7 software. (**b**) Ms-cd1-GFP is localized in nucleus. Marker represents a known marker protein localized in nucleus. Scale bars, 30 μm. Experiments were repeated three times independently with similar results.

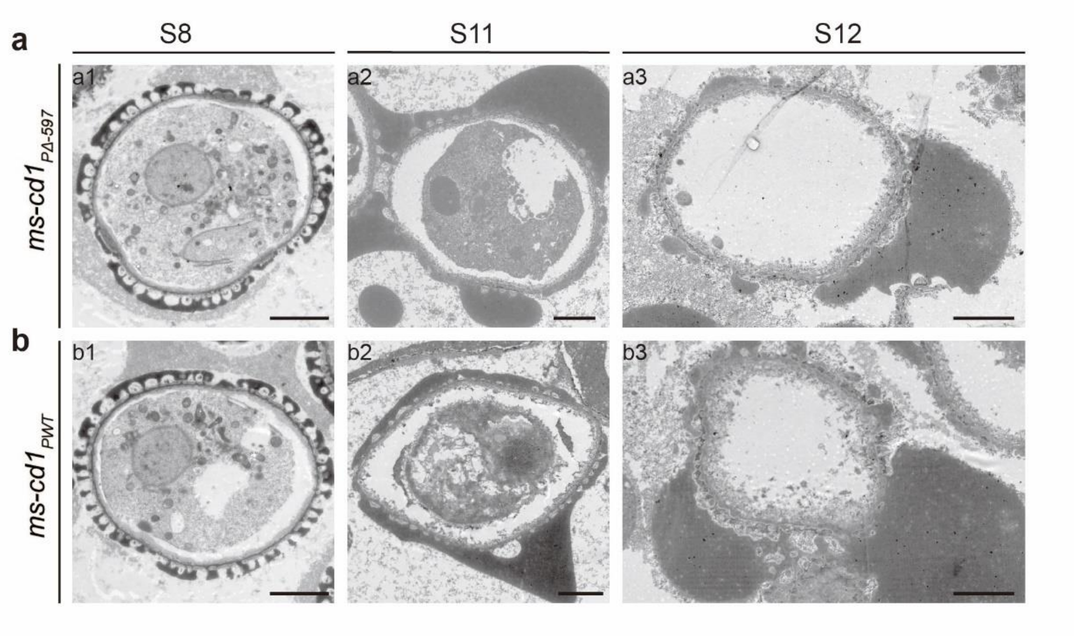

Supplementary Figure 7. TEM analysis of microspores in the *ms-cd1$_{PWT}$* and *ms-cd1$_{P\Delta\text{-}597}$* mutants at anther development stages S8, S11 and S12. (a) Representative microspores at stages S8, S11 and S12 in *ms-cd1$_{P\Delta\text{-}597}$* mutant. **(b)** Representative microspores at stages S8, S11 and S12 in *ms-cd1$_{PWT}$* mutant. Scale bars, 3 μm. Experiments were repeated three times independently with similar results.

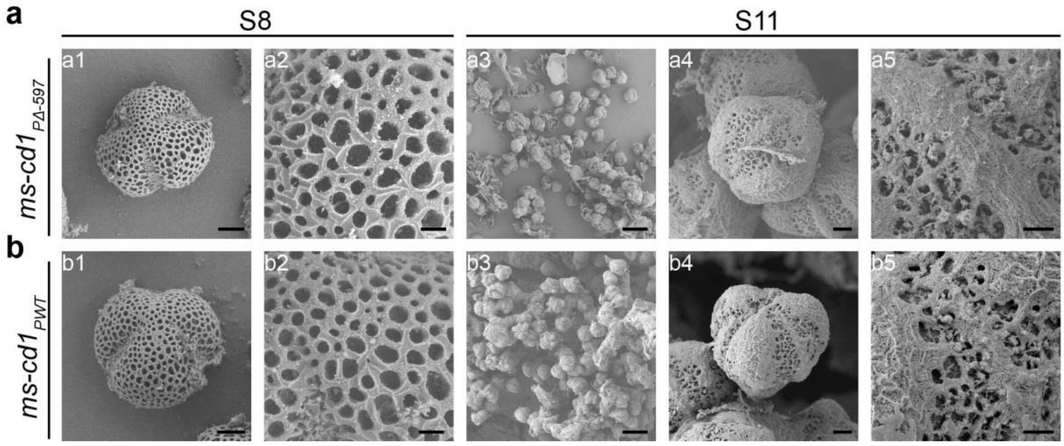

Supplementary Figure 8. SEM analysis of microspores in the *ms-cd1*$_{PWT}$ and *ms-cd1*$_{P\Delta\text{-}597}$ mutants at anther development stages S8 and S11. (a) Representative microspores at stages S8 and S11 in *ms-cd1*$_{P\Delta\text{-}597}$ mutant. **(b)** Representative microspores at stages S8 and S11 in *ms-cd1*$_{PWT}$ mutant. Scale bars, 3 µm in a1, a4, b1 and b4, 1 µm in a2, a5, b2 and b5, 25 µm in a3 and b3. Experiments were repeated three times independently with similar results.

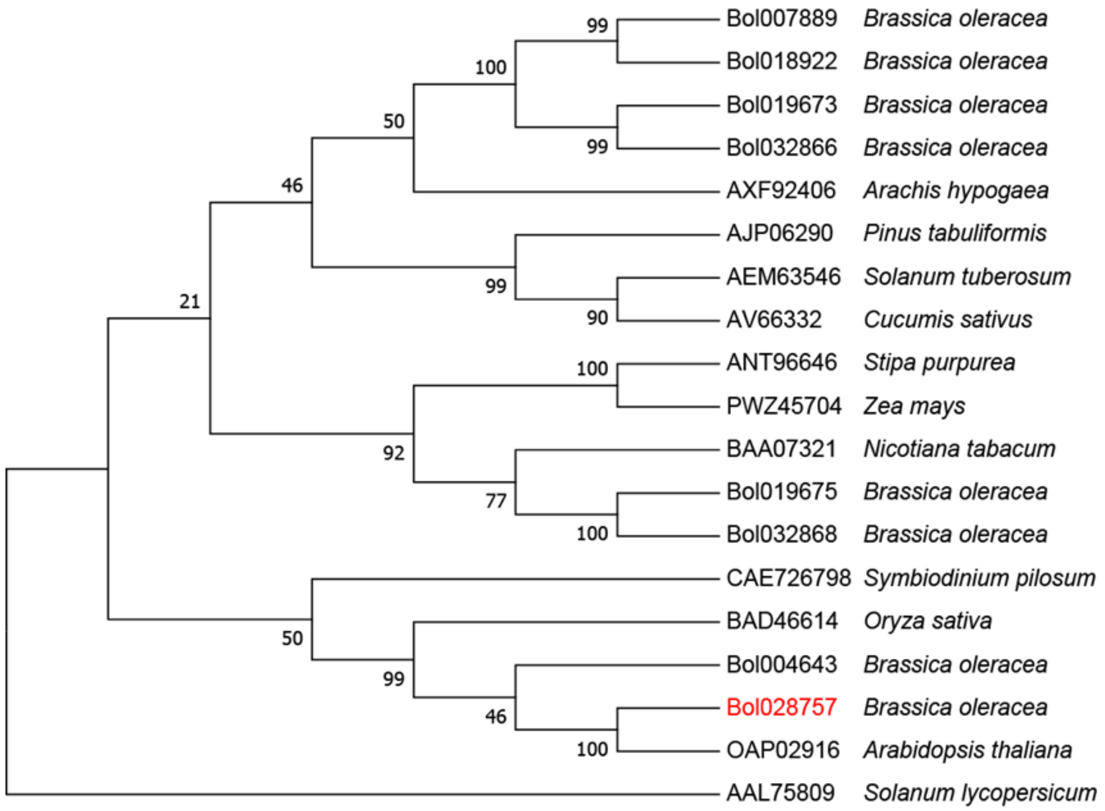

Supplementary Figure 9. Phylogenetic analysis of BoERF1L. BoERF1L (Bol028757) is indicated in red. The neighbour-joining phylogenetic tree (1000 bootstrap replications) was constructed using MEGA7 software.

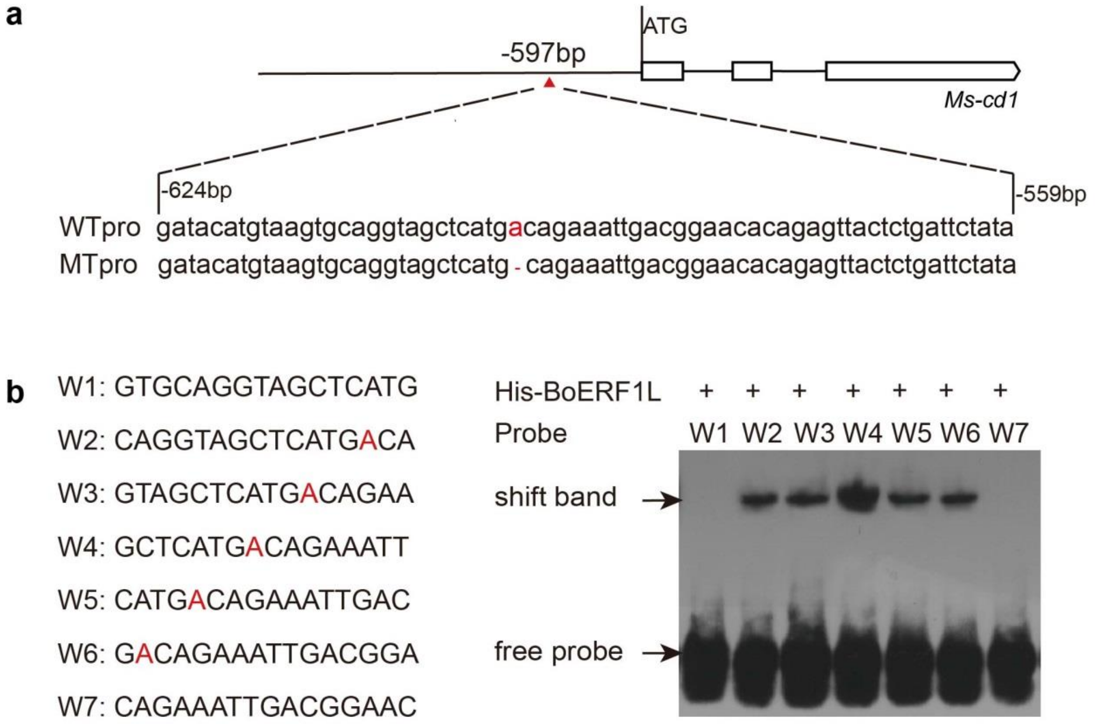

Supplementary Figure 10. EMSA analyses. (**a**) The probes WTpro and MTpro used for EMSA assays. (**b**) Seven short probes from WTpro and EMSA analyses for them. W1 and W7 lose the binding ability to His-BoERF1L; W2, W3, W5 and W6 have reduced binding; and W4 is not affected in binding. Experiments were repeated three times independently with similar results. Source data are provided as a Source Data file.

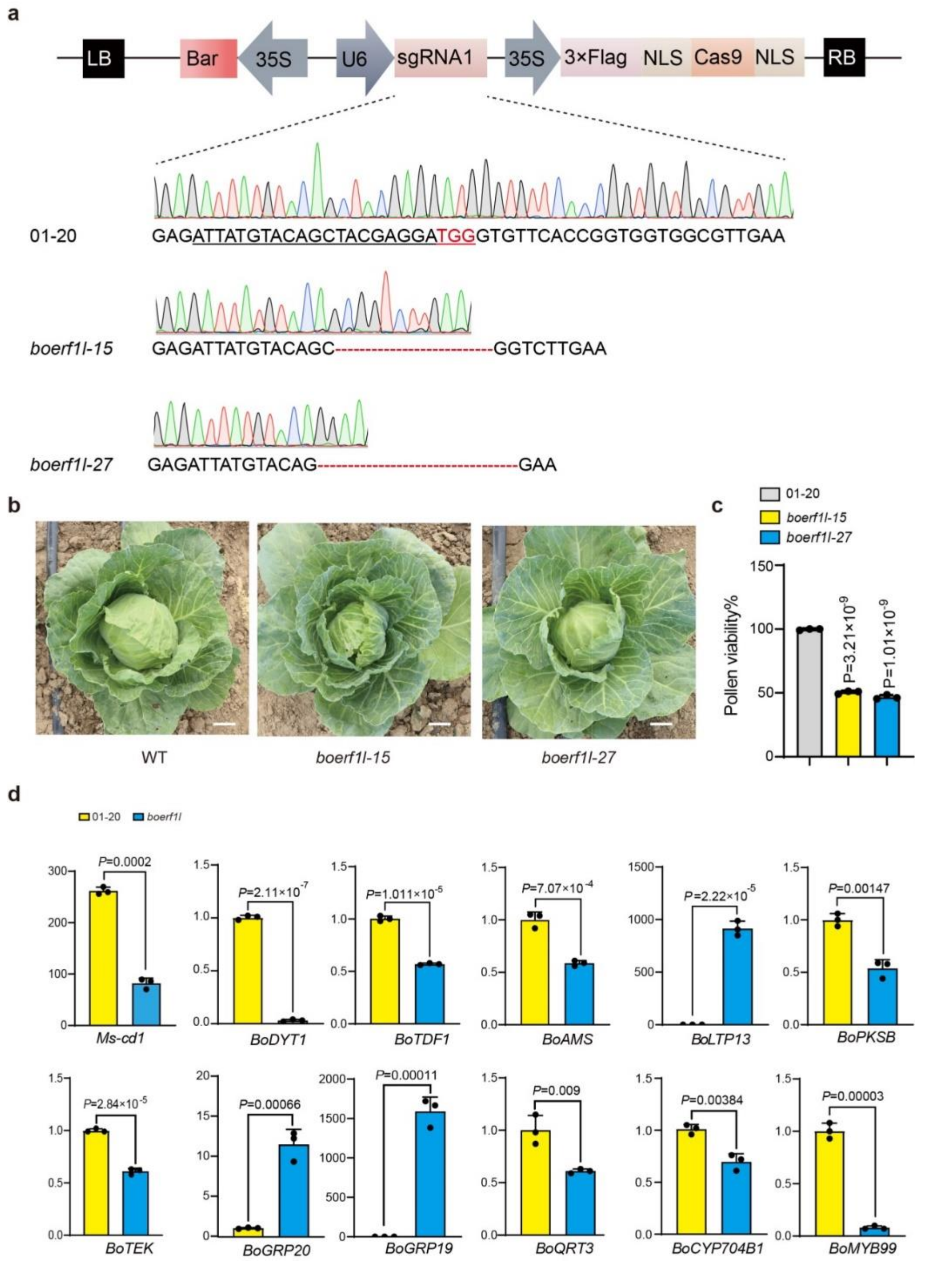

Supplementary Figure 11. Creation and characterization of *boerf1l* mutants. (a) Schematic diagram of the CRISPR/Cas9 construct targeting *BoERF1L* and DNA sequence analysis of the sgRNA targeted site among WT and the knock-out lines generated by CRISPR/Cas9 system. U6, *Arabidopsis* U6-26 promoter; sgRNA1, a single guide RNA targeting *BoERF1L.* Mutations are indicated in red. **(b)** Comparation of whole plant phenotypes of 01-20 and two *boerf1l* mutants at heading stage. Scale bar, 5 cm. **(c)** Statistical analysis of pollen viability among WT and two *boerf1l* mutant plants. **(d)** The gene expression in 01-20 and *boerf1l* mutant. qRT-PCR analysis of *Ms-cd1* and its closely related genes associated with pollen development in 01-20 and *boerf1l* mutant anthers. Data are presented as means ± SD, (n = 3 for c and d). Two-tailed unpaired *t*-test was used for statistical analysis. Experiments were repeated three times independently with similar results. Source data are provided as a Source Data file.

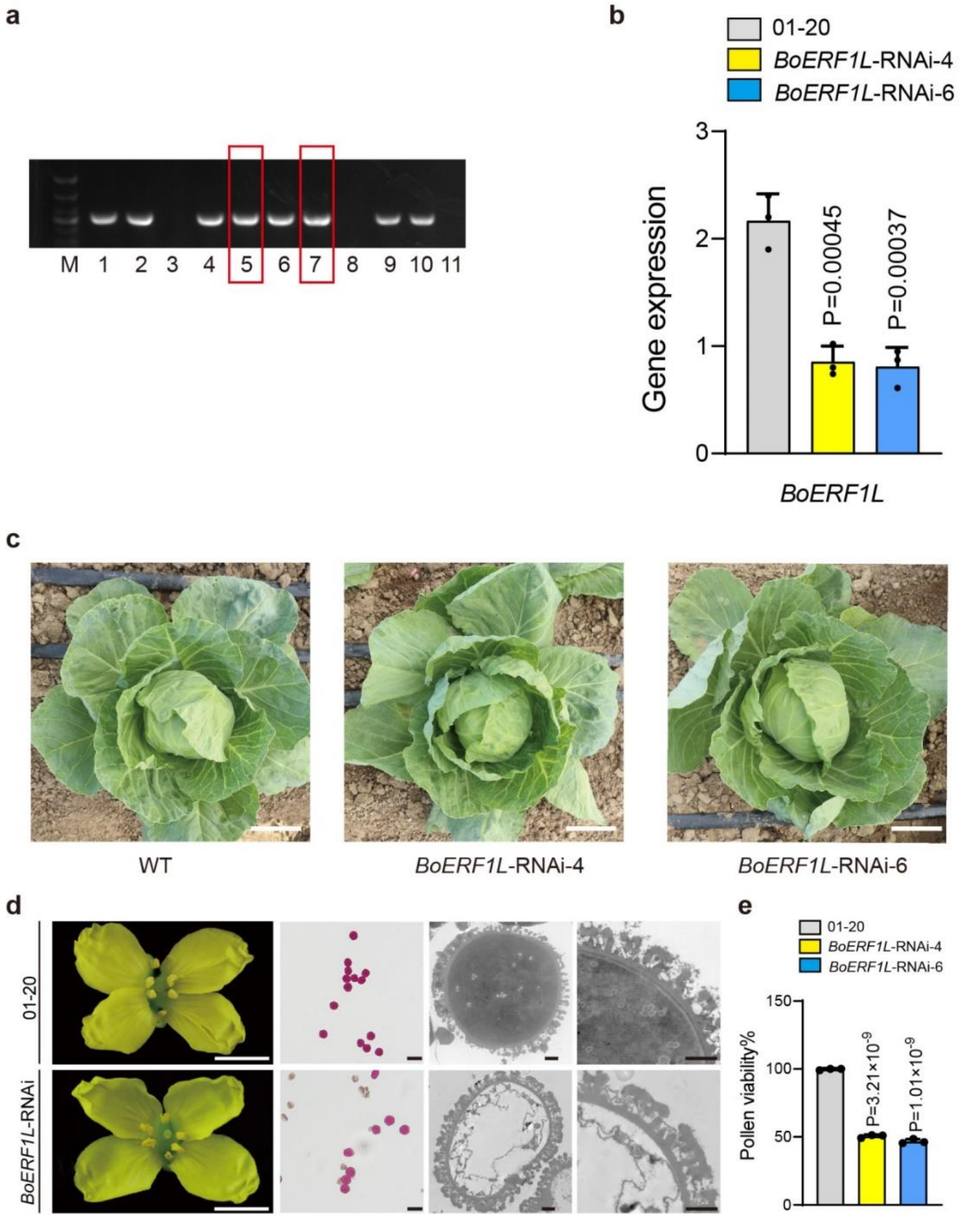

Supplementary Figure 12. Characterization of *BoERF1L-RNAi* plants. (a) PCR confirmation of *BoERF1L-RNAi* positive plants using specific primers for *Bar* gene. M, DNA maker ladders. Lanes 1-11, 11 detected plants, representative plants *BoERF1L-RNAi-4* (lane 5) and *BoERF1L-RNAi-6* (lane 7) are marked in red blocks. **(b)** The relative expression levels of *BoERF1L* in WT and *BoERF1L-RNAi* plants**. (c)** Comparation of whole plant phenotypes of 01-20 and *BoERF1L-RNAi* plants at heading stage. Scale bar, 5 cm. **(d)** Flowers, anthers, pollen grains stained with Alexander solution and TEM analysis of microspore among WT and *BoERF1L* RNA interference plants. Scale bar, 5 mm in A1 and A5, 50 μm in A2 and A6, 5 μm in A3 and A7, 2 μm in A4 and A8. **(e)** Statistical analysis of pollen viability among WT and *BoERF1L* RNA interference plants. Data are presented as means ± SD (n = 3 for b and e). Two-tailed unpaired *t*-test was used for statistical analysis. Experiments were repeated three times independently with similar results. Source data are provided as a Source Data file.

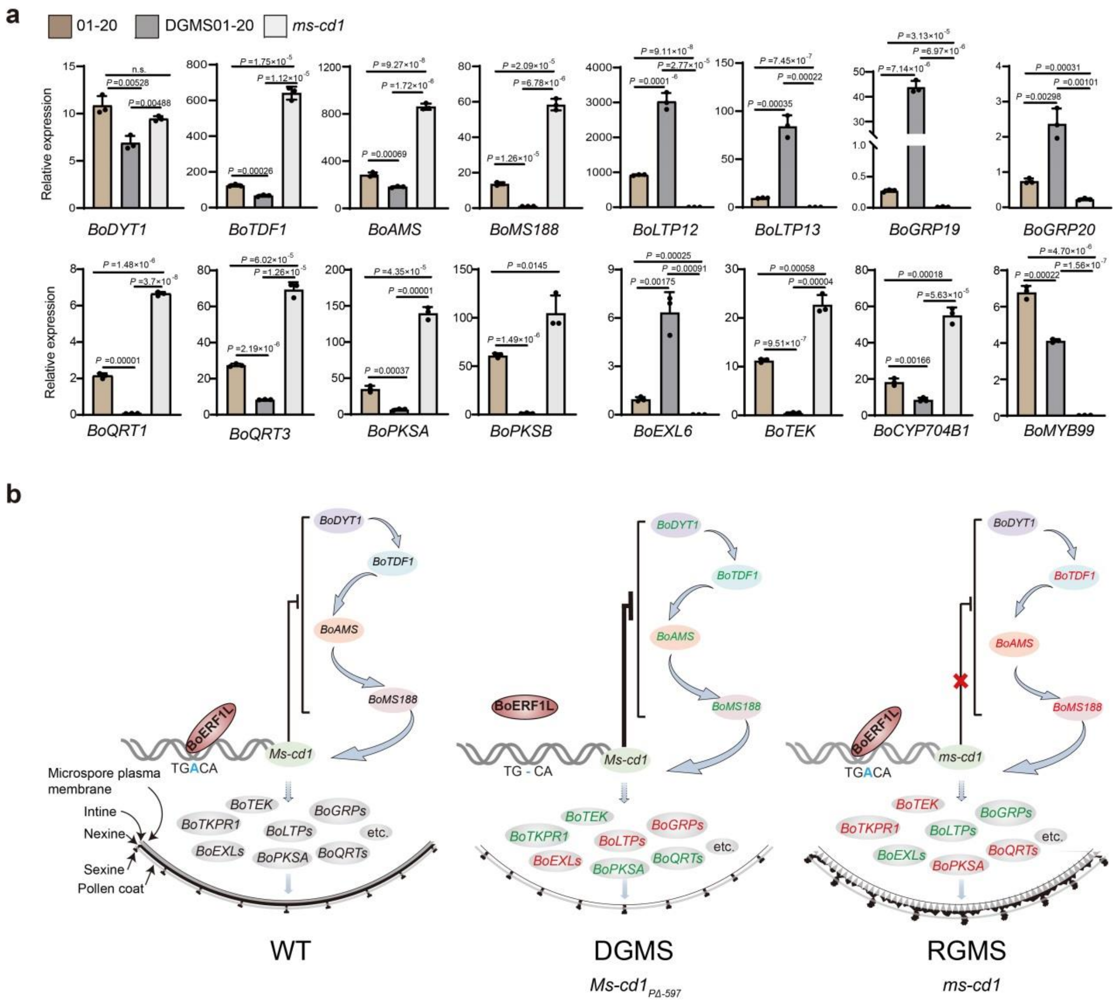

Supplementary Figure 13. *Ms-cd1*$_{P\Delta\text{-}597}$ and loss of function of *Ms-cd1* alters expression of genes required for tapetum and pollen development. (a) qRT-PCR analysis of 16 representative genes associated with pollen development in WT, DGMS01-20 and *ms-cd1* mutant anthers at stages 6 to 9. **(b)** Model of *Ms-cd1* controlling male fertility in dominant and recessive manners. Arrows indicates positive regulation, black lines indicate inhibition, bold black lines indicate enhanced inhibition, red × indicates disrupted feedback regulation, genes/proteins in red color indicates upregulation, and that in green color indicate downregulation. Data are presented as means ± SD, (n = 3 for a). Two-tailed unpaired *t*-test was used for statistical analysis. Experiments were repeated three times independently with similar results. Source data are provided as a Source Data file.

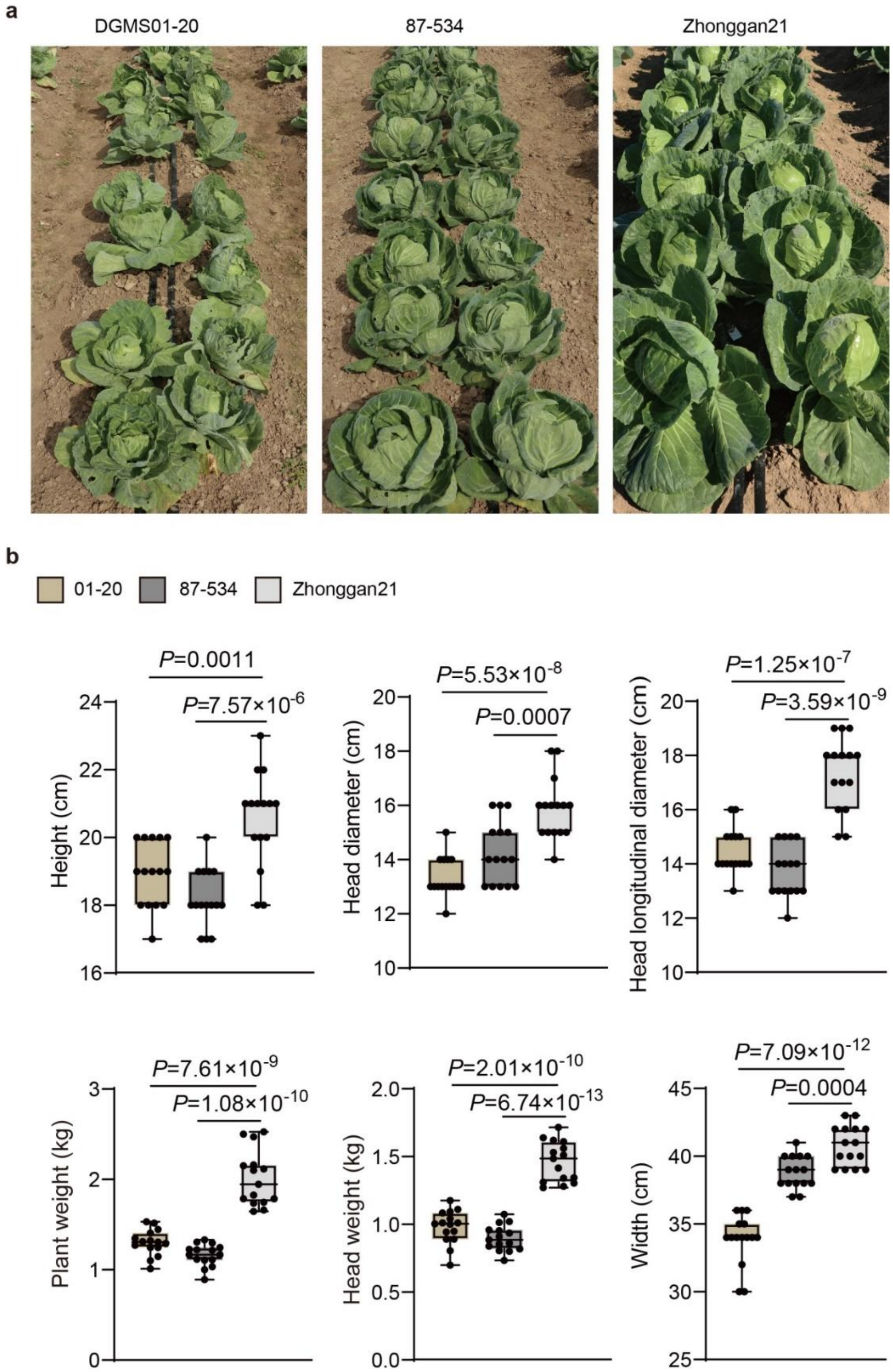

Supplementary Figure 14. Elite hybrid Zhonggan21 produced via DGMS-based system. (a) The phenotypes of DGMS01-20, 87-534 and Zhonggan21. **(b)** Statistical analysis of 6 traits showed that Hybrid Zhonggan21 had obvious heterosis comparing with its parental lines DGMS01-20 and 87-534. Data are presented as means ± SD, n = 15. Two-tailed unpaired *t*-test was used for statistical analysis. Experiments were repeated three times independently with similar results. Source data are provided as a Source Data file.

Supplementary Table 1. ***79-399-3*** **derived dominant male sterile mutant is controlled by a single gene.**

**Plant materials**	**Total plants number**	**Observed**		**Segregation ratio (F:S)**	**Expected ratio (F:S)**	χ^2	***P* value**
		**Fertile plants**	**Sterile plants**				
HO-DGMS01-20 × 01-20	50	0	50	-	-	-	
HE-DGMS01-20 × 01-20	2660	1363	1297	1.05 : 1	1:1	1.64	0.20
HO-DGMS87-534 × 87-534	49	0	49	-	-	-	
HE-DGMS87-534 × 87-534	2284	1155	1129	1.02 : 1	1:1	0.30	0.59
HE-DGMS18K × 18K	3832	1959	1873	1.05 : 1	1:1	1.93	0.16

Chi-Square goodness of fit test is used.

Supplementary Table 2. Inheritance of male phenotype of *PΔ-597::gMs-cd1* transgenic plants, *ms-cd1$_{PWT}$* and *ms-cd1$_{PΔ-597}$* mutants.

**Plant materials**	**Total plants**	**Male fertility**	**Male sterility**	**Expected ratio**	χ^2	***P* value**	**Inheritance**
01-20	40	40	0	-	-		-
PΔ-597::gMs-cd1 T0-1 × 01-20	40	20	20	1:1	0	1	dominant
PΔ-597::gMs-cd1 T0-3 × 01-20	40	21	19	1:1	0.1	0.75	dominant
ms-cd1$_{PWT}$-5 × 01-20 (F_1)	20	20	0	-	-		-
ms-cd1$_{PWT}$-5 × 01-20 (F_2)	40	31	9	3:1	0.13	0.72	recessive
ms-cd1$_{PWT}$-7 × 01-20 (F_1)	20	20	0	-	-		-
ms-cd1$_{PWT}$-7 × 01-20 (F_2)	40	32	8	3:1	0.53	0.47	recessive
ms-cd1$_{PΔ-597}$-1 × 01-20 (F_1)	20	20	0	-	-		-
ms-cd1$_{PΔ-597}$-1 × 01-20 (F_2)	40	30	10	3:1	0	1	recessive
ms-cd1$_{PΔ-597}$-4 × 01-20 (F_1)	20	20	0	-	-		-
ms-cd1$_{PΔ-597}$-4 × 01-20 (F_2)	40	31	9	3:1	0.13	0.72	recessive

Chi-Square goodness of fit test is used.

Supplementary Table 3. Positive clones from yeast-one hybrid assays.

Gene ID	Annotations	Frequency
Bol028757	ERF1, transcription factor	9/14
Bol006547	PSAO; PSAO (photosystem I subunit O)	1/14
Bol043847	aspartic-type endopeptidase	1/14
Bol019677	UCH3, ubiquitin thiolesterase	1/14
Bo5g052280	Glycine-rich protein family	1/14

Supplementary Table 4. *Ms-cd1$_{P\Delta\text{-}597}$* confers dominant male sterility in *Arabidopsis*, rice, *Brassica napus* and tomato.

Cross combination	Total plants	Fertility	Sterility	Expected ratio	χ^2	*P* value	Inheritance
PΔ-597::Ms-cd1-At-3 × *Col-0*	20	13	7	1:1	1.8	0.18	dominant
PΔ-597::Ms-cd1-At-4 × *Col-0*	20	12	8	1:1	0.8	0.37	dominant
PΔ-597::Ms-cd1-Os-1 × ZH11	20	14	6	1:1	3.2	0.07	dominant
PΔ-597::Ms-cd1-Os-12 × ZH11	20	8	12	1:1	0.8	0.37	dominant
PΔ-597::Ms-cd1-Bn-7 × Westar	20	13	7	1:1	1.8	0.18	dominant
PΔ-597::Ms-cd1-Bn-9 × Westar	20	7	13	1:1	1.8	0.18	dominant
PΔ-597::Ms-cd1-Sl-9 × AC	20	12	8	1:1	0.8	0.37	dominant
PΔ-597::Ms-cd1-Sl-13 × AC	20	9	11	1:1	0.2	0.65	dominant

Chi-Square goodness of fit test is used.
