# Supplementary material for: A natural mutation in the promoter of Ms-cd1 causes dominant male sterility in Brassica oleracea
Source: Nat Commun. 2023 Oct 5;14:6212. doi: 10.1038/s41467-023-41916-0 (PMC10556095; doi:10.1038/s41467-023-41916-0)
Supplement: Supplementary file 3 — Description of Additional Supplementary Files [file 41467_2023_41916_MOESM3_ESM.pdf]

Description of Additional Supplementary Files

File Name: Supplementary Data 1
Description: Materials developed and used in this study.

File Name: Supplementary Data 2
Description: Primers used in this study.

File Name: Supplementary Data 3
Description: Ms-cd1 homologues in 13 species.

File Name: Supplementary Data 4
Description: Sequences of the *Ms-cd1*$_{PWT}$ and *Ms-cd1*$_{P\Delta\text{-}597}$.
