# Supplementary material for: A natural mutation in the promoter of Ms-cd1 causes dominant male sterility in Brassica oleracea
Source: Nat Commun. 2023 Oct 5;14:6212. doi: 10.1038/s41467-023-41916-0 (PMC10556095; doi:10.1038/s41467-023-41916-0)
Supplement: Supplementary file 8 — Reporting Summary [file 41467_2023_41916_MOESM8_ESM.pdf]

nature portfolio

Corresponding author(s): DAPR NCOMMS-23-13851A

Last updated by author(s): Sep 7, 2023

Reporting Summary

Nature Portfolio wishes to improve the reproducibility of the work that we publish. This form provides structure for consistency and transparency in reporting. For further information on Nature Portfolio policies, see our Editorial Policies and the Editorial Policy Checklist.

Statistics

For all statistical analyses, confirm that the following items are present in the figure legend, table legend, main text, or Methods section.

n/a	Confirmed	
☐	☒	The exact sample size (*n*) for each experimental group/condition, given as a discrete number and unit of measurement
☐	☒	A statement on whether measurements were taken from distinct samples or whether the same sample was measured repeatedly
☐	☒	The statistical test(s) used AND whether they are one- or two-sided<br>*Only common tests should be described solely by name; describe more complex techniques in the Methods section.*
☒	☐	A description of all covariates tested
☒	☐	A description of any assumptions or corrections, such as tests of normality and adjustment for multiple comparisons
☐	☒	A full description of the statistical parameters including central tendency (e.g. means) or other basic estimates (e.g. regression coefficient) AND variation (e.g. standard deviation) or associated estimates of uncertainty (e.g. confidence intervals)
☐	☒	For null hypothesis testing, the test statistic (e.g. *F*, *t*, *r*) with confidence intervals, effect sizes, degrees of freedom and *P* value noted<br>*Give P values as exact values whenever suitable.*
☒	☐	For Bayesian analysis, information on the choice of priors and Markov chain Monte Carlo settings
☒	☐	For hierarchical and complex designs, identification of the appropriate level for tests and full reporting of outcomes
☒	☐	Estimates of effect sizes (e.g. Cohen's *d*, Pearson's *r*), indicating how they were calculated

Our web collection on statistics for biologists contains articles on many of the points above.

Software and code

Policy information about availability of computer code

Data collection	In this study,no custom-made software or codes/mathematical algorithms were generated by the authors. Pollen grains were imaged with a BX-53TR microscope (Olympus, Tokyo, Japan). Plant tissues were captured with a Canon EOS M6 digital camera (Canon, Tokyo, Japan) or SZX2-ILLB16 stereomicroscope (Olympus, Tokyo, Japan). GFP signal was detected using a confocal microscope (Carl Zeiss AG, Oberkochen, Germany). The luciferase luminescence was captured using a Tanon 5200 system (Berthold, Tanon, Shanghai, China).
Data analysis	Statistical analyses were conducted using GraphPad Prism (Version 9). Phylogenetic tree was constructed with MEGA software (version 7). Images were processed with Adobe Illustrator 2022 (version 26.2) and Adobe Photoshop CS6 (version 13.0).

For manuscripts utilizing custom algorithms or software that are central to the research but not yet described in published literature, software must be made available to editors and reviewers. We strongly encourage code deposition in a community repository (e.g. GitHub). See the Nature Portfolio guidelines for submitting code & software for further information.

Data

Policy information about availability of data

All manuscripts must include a data availability statement. This statement should provide the following information, where applicable:

- Accession codes, unique identifiers, or web links for publicly available datasets
- A description of any restrictions on data availability
- For clinical datasets or third party data, please ensure that the statement adheres to our policy

The genomic resequencing data have been deposited in the NCBI Sequence Read Archive under accession number PRJNA1013233. The sequence data for Ms-cd1

have been deposited in GenBank under accession number BankIt2741573 (OR523690 and OR523691). Sequence for BoERF1L can be found in Brassica Database (http://brassicadb.cn). Other data supporting the findings of this study are available within the article and its Supplementary Information files. The primers used in this study are provided as Supplementary Data 2. Source data are provided with this paper.

Research involving human participants, their data, or biological material

Policy information about studies with human participants or human data. See also policy information about sex, gender (identity/presentation), and sexual orientation and race, ethnicity and racism.

Reporting on sex and gender	n/a
Reporting on race, ethnicity, or other socially relevant groupings	n/a
Population characteristics	n/a
Recruitment	n/a
Ethics oversight	n/a

Note that full information on the approval of the study protocol must also be provided in the manuscript.

Field-specific reporting

Please select the one below that is the best fit for your research. If you are not sure, read the appropriate sections before making your selection.

- [x] Life sciences
- [ ] Behavioural & social sciences
- [ ] Ecological, evolutionary & environmental sciences

For a reference copy of the document with all sections, see nature.com/documents/nr-reporting-summary-flat.pdf

Life sciences study design

All studies must disclose on these points even when the disclosure is negative.

Sample size	We follow general knowledge, instructions for standard protocols and related studies to determine the sample size.<br>For plant phenotypes, three or more individual plants were analyzed.<br>For qRT-PCR: at least 100 mg plant tissues were pooled together and extracted total RNA. At least three individual qRT-PCRs were performed.<br>For pollen fertility analysis: At least three lines with three biological replicates were performed.<br>For Y1H assays, about 2 g cabbage floral buds (< 3.5 mm) were collected for cDNA library construction.<br>For Dual-LUC transient expression assays, at least three tobacco plants for each replicate were used sampled.
Data exclusions	No data was excluded from the analyses
Replication	We conducted at least three biological replicates for each experiment. Some experiments, including microscopic images collection, qRT-PCR, Dul-luc, etc. were repeated three times independently with similar results. These information has been described in the methods or relevant figure legends.
Randomization	Plants, including cabbage, A. thaliana, N. benthamiana etc. used in this study were randomly grown in the same open field, greenhouse or growth chamber. Plants samples were collected randomly.
Blinding	Researchers were blinded during microscopic images collection including SEM, TEM, dual-LUC analysis, and some other assays including EMSA and Y1H.

Reporting for specific materials, systems and methods

We require information from authors about some types of materials, experimental systems and methods used in many studies. Here, indicate whether each material, system or method listed is relevant to your study. If you are not sure if a list item applies to your research, read the appropriate section before selecting a response.

Materials & experimental systems

n/a	Involved in the study
[x]	[ ] Antibodies
[x]	[ ] Eukaryotic cell lines
[x]	[ ] Palaeontology and archaeology
[x]	[ ] Animals and other organisms
[x]	[ ] Clinical data
[x]	[ ] Dual use research of concern
[ ]	[x] Plants

Methods

n/a	Involved in the study
[x]	[ ] ChIP-seq
[x]	[ ] Flow cytometry
[x]	[ ] MRI-based neuroimaging

Dual use research of concern

Policy information about dual use research of concern

Hazards

Could the accidental, deliberate or reckless misuse of agents or technologies generated in the work, or the application of information presented in the manuscript, pose a threat to:

No	Yes
[ ]	[ ] Public health
[ ]	[ ] National security
[ ]	[ ] Crops and/or livestock
[ ]	[ ] Ecosystems
[ ]	[ ] Any other significant area

Experiments of concern

Does the work involve any of these experiments of concern:

No	Yes
[ ]	[ ] Demonstrate how to render a vaccine ineffective
[ ]	[ ] Confer resistance to therapeutically useful antibiotics or antiviral agents
[ ]	[ ] Enhance the virulence of a pathogen or render a nonpathogen virulent
[ ]	[ ] Increase transmissibility of a pathogen
[ ]	[ ] Alter the host range of a pathogen
[ ]	[ ] Enable evasion of diagnostic/detection modalities
[ ]	[ ] Enable the weaponization of a biological agent or toxin
[ ]	[ ] Any other potentially harmful combination of experiments and agents
